# Evaluation of warning strategies to reduce faking during military recruitment

**DOI:** 10.1080/08995605.2023.2243364

**Published:** 2023-08-28

**Authors:** Justin Ryan Feeney, Richard D. Goffin, Colin Kemp, Shadi Beshai, Joy D. Klammer

**Affiliations:** a School of Business, Rhode Island College, Providence, Rhode Island; b Department of Psychology, University of Western Ontario, London, Ontario, Canada; c Director General Military Personnel Research and Analysis, Department of National Defence (Canada), Ottawa, Ontario, Canada; d Department of Psychology, University of Regina, Regina, Saskatchewan, Canada

**Keywords:** Personality assessment, job applications, faking, faking warnings, military, recruitment

## Abstract

The applicant faking literature suggests that faking warnings – brief messages that dissuade applicants from faking – can reduce faking on personality tests by up to 50%. However, the efficacy of warnings may be limited by their atheoretical construction. Further, these threatening messages can cause applicants to feel negatively about the personality test, potentially reducing their validity during the selection process. We tried to improve the efficacy of faking warnings, while minimizing negative applicant reactions, by leveraging theory from the accountability and morality literatures. We tested three new faking warnings that emphasized short-term accountability, long-term accountability, and morality. To do so, we tested 466 military trainees undergoing basic training at the Canadian Armed Forces and asked them to engage in a selection simulation. We assigned groups of trainees to the different faking warning conditions and guided them through the simulation. We found that a faking warning emphasizing short-term accountability, which threatened to detect fakers by contacting references and using “internal integrity checks,” reduced applicant faking. None of the other messages had any effect when compared to a no-warning control group.

**What is the public significance of this article?—**Military (as well as civilian organizations) often use personality assessments during their recruitment and selection processes. One major concern in with these assessments across contexts is that job candidates often fake – providing the answers they think their prospective employer wants to see rather than the truest response. Using a simulation, we found that warning military trainees that faking can be detected and will lead to disqualification, provided modest reductions in faking. We also found that appealing to trainee’s long-term interests or trying to morally assuage trainees had no effect on faking.

Imagine you are being considered for a coveted job – one that has everything you want – and the hiring company requests that you complete a personality test. The test asks about your punctuality – a trait that you know is highly desirable, but one that is your personal weakness. Do you answer honestly or lie? Job applications generate incentive for applicants to “fake” a more competitive profile than warranted during the selection process. Faking has been defined as applicants deliberately providing “inaccurate responses to personality items in a manner that they believe will increase their chances of obtaining valued outcomes, such as a favorable hiring decision” (Goffin & Boyd, 2009, p. 151)

There is compelling evidence that a large percentage of job applicants fake their responses to personality tests (Donovan et al., 2013; Holden et al., 2017), and this faking reduces the validity of personality tests. For example, personality tests exhibit lower self-peer correlations within samples where applicants are faking when compared to samples where applicants are not faking (Robie et al., 2009). In addition, faking has been shown to reduce the criterion-related validity of personality tests (Peterson et al., 2011). Reductions in validity may have undesirable consequences for organizations by reducing the accuracy of hiring decisions.

The purpose of this study was to examine the extent that faking warnings could reduce faking on personality testing by applicants during the military hiring process. We developed three faking warnings that leveraged accountability (Lerner & Tetlock, 1999) and morality theory (Haidt, 2001) with the intent of improving selection decisions. Similar to civilian jobs, personality testing predicts important outcomes in military settings, including task performance, counterproductive work behavior, and leadership training (Bartone et al., 2002; Darr, 2011; Darr & Catano, 2016) and is used in their initial recruitment. Not surprisingly then, *some* military applicants fake during the selection stage to look more favorable on these measures. In a study of applicants to the Swiss Armed Forces (Boss et al., 2015), self-reported military service motivation predicted Conscientiousness (*r* = .50) and Extraversion (*r* = .45) scores during initial recruitment, and these were highest among those who admitted to “faking good.” In another study using a simulated military induction, military applicants were most likely to fake on the personality measures of Conscientiousness, Emotional Stability, and Extraversion (Holden & Book, 2009).

### Reducing faking

Several methods exist to mitigate the negative outcomes of applicant faking (Griffith & Robie, 2013). One popular technique is to include faking warnings, messages placed prior to or during the personality test, to dissuade individuals from faking on the test. The current best practice is to inform applicants that faking can be detected and that detection will lead to immediate disqualification from the selection process (Dwight & Donovan, 2003). Several studies suggest that faking warnings may reduce the extent that each applicant fakes by between 30% and 50% (Fan et al., 2012; Landers et al., 2011; Robson et al., 2008). That being said, there are some studies that suggest faking warnings are much less effective in reducing faking (Fisher et al., 2018; Vasilopoulos et al., 2005). For the purposes of this study, we use a Traditional Faking Warning that informs applicants that responses will be verified, and that dishonesty can lead to disqualification. This wording is based on the faking warning currently used by the Canadian Armed Forces.

Scholars have suggested that we may able to improve faking warnings by leveraging psychological theory (Goffin & Boyd, 2009). Accordingly, we examined if we could bolster the efficacy of faking warnings by crafting them to incorporate core ideas from accountability theory (Lerner & Tetlock, 1999). The accountability literature revealed two methods that might enhance the potency of faking warnings: applicants should feel more accountable when they believe (a) there is a credible process in place to check the validity of their answers; and (b) they will need to defend their answers on an item-by-item basis – rather than just their overall score.

We designed two faking warnings that increased accountability by describing a credible process for personality verification during the application process. The goal was to make applicants to the military focus on the process of responding to the test, rather than the outcome (Feeney et al., 2023; Lerner & Tetlock, 1999). The Immediate Authentication Warning informed applicants that the personality test contained test items that could detect faking and provided examples from an impression management scale (Blasberg et al., 2013). The Immediate Authentication Warning also informed applicants that their responses to personality items could be verified by calling references that they provided during initial recruitment. Immediate Authentication Warning also informed applicants that if they were identified as faking, they would need to defend their responses to each personality item to a trained Recruiting Officer from the Canadian Armed Forces. By having applicants consider if they could defend their answers to each personality item, in theory, they should fake less in cases where they would struggle to defend their choices.

**H_1_**: The Immediate Authentication Warning will reduce personality faking when compared to a traditional faking warning and a no-warning control group.

The Future Authentication Warning capitalized on the same accountability mechanisms as the Immediate Authentication Warning, but it attempted to dissuade applicants to the military from faking by focusing on future consequences. The Future Authentication Warning explained to applicants that their Recruitment Officer would compare their observed personality during training to their answers on the personality test. The Future Authentication Warning attempted to build credibility by illustrating that the personality test assessed behaviors that can be verified by observing them. The Future Authentication Warning explained that any applicant identified as faking would need to defend their responses to each personality item to the Recruitment Officer. Applicants were told that failure to do so would lead to disqualification from the selection process. This feature also aimed to prompt applicants to carefully consider if they could defend their answers to each personality item rather than their overall test scores. Finally, the Future Authentication Warning also included an educational component. It explained to applicants that their personality scores would be used for placement decisions and that faking may lead to placement in a position that was a poor fit for them. We explained that being placed in a position with poor fit would lead to lower performance, and in turn, fewer opportunities for promotion.

**H_2_**: The Future Authentication Warning will reduce personality faking when compared to a traditional faking warning and a no-warning control group.

In contrast to threats of faking detection, scholars have also suggested appealing to the test-takers’ morality to reduce applicant faking (Feeney et al., 2023; Goffin & Boyd, 2009; Robie et al., 2007). Goffin and Boyd (2009, p. 158) suggested that “test-taking instructions that appeal to the test takers’ moral compass (by emphasizing that faking is a form of lying or cheating that violates most accepted standards of moral behavior) might add to the success of existing faking warnings.” Consistent with this suggestion, Uruena and Robie (2011, p. 17) found that applicants who read the test instructions “dishonest or distorted self-descriptions are simply wrong and do not adhere to commonly accepted standards of behavior” scored lower on a measure of conscientiousness than applicants who did not read them (*d* = .23).

To enhance the efficacy of this approach, we created a Moral Suasion Appeal that utilizes theoretical work on morality, and principles from the persuasion literature, to increase its salience with applicants. Our tailored Moral Suasion Appeal nstructed applicants to consider a life-threatening combat attack and imagine how they would feel if an intelligence officer had information about that attack and forgot to relay the information to their commander; not from malice, but carelessness – because that person was placed into their position as a result of faking. The goal of the Moral Suasion Appeal was to prompt applicants to feel negatively about faking, and in turn, have them answer more honestly. Moral decisions are generally informed by affective responses rather than rational decision-making (Haidt, 2001). Thus, we also included visuals to facilitate thinking about the combat scenario to maximize the emotional response to the simulation (Lang et al., 1993; Schimmack, 2005). We include a summary of our faking warning conditions in Table 1 below.

**H_3_**: The Moral Suasion Appeal will reduce personality faking when compared to a traditional faking warning and a no-warning control group.

**Table 1.** Faking warning summary.

Name	Key Features
Traditional Faking Warning	A short warning that informs applicants that responses will be verified, and dishonesty will lead to disqualification.
Immediate Authentication Warning	A descriptive warning that tries to appeal to applicants’ short-term interests by asserting that the personality test includes items that can detect faking, and that faking could be verified by calling references. It also asserts that faking may lead to needing to defend responses to an officer.
Future Authentication Warning	A descriptive warning that tries to appeal to applicants’ long-term interests by asserting that faking may be apparent sometime in the future and that placement based on faked scores could lead to a less successful career.
Moral Suasion Appeal	A message that tries to persuade applicants to feel negative about faking by having them imagine the negative consequences of working with an officer who was recruited due to faking.
No Faking Warning	No warning is present. Condition serves as a baseline for comparison.

### Candidate reactions to faking warnings

Faking warnings that threaten applicants can have the unintended consequence of increasing test-taking anxiety (Converse et al., 2008). In addition, warnings increase the difficulty of filling out the personality test – especially for those with low general mental ability (Vasilopoulos et al., 2005) – and therefore, applicants may form adverse reactions about the fairness or appropriateness of the test. Applicant reactions are essential because applicants crystallize negative impressions about the organization, which could, in turn, facilitate undesired outcomes such as discouraging others from applying to the organization (Feeney et al., 2015, 2023; McCarthy et al., 2009). Applicant reactions are critical because negative impressions can reduce the likelihood an applicant accepts job offers or recommends the organization to others (McCarthy, Bauer, Truxillo Anderson et al., 2017; McCarthy, Bauer, Truxillo, Campion et al., 2017).

Both the Immediate Authentication Warning and Future Authentication Warning threaten applicants with multiple sources of verification and may instigate higher levels of test-taking anxiety and negative perceptions of procedural justice. For example, applicants may think it is unfair to use their references or on-the-job performance to verify their personality responses, or become anxious about future detection. By contrast, our Moral Suasion Appeal may lead to less anxiety and more favorable perceptions of procedural justice, because it omits threatening language. That being said, honest respondents might find the threats in the Immediate Authentication Warning or Future Authentication Warning reassuring, because they may believe that fakers will be detected and punished, making the personality test fairer and more accurate. Similarly, the Moral Suasion Appeal may also alert applicants that their competition is faking and does not provide any corrective mechanism, which could weaken perceptions of accuracy and fairness. As a result, it is difficult to draw clear hypotheses of how the new faking warnings will affect applicant reactions. This investigation presents a novel contribution to the literature because few studies have examined how faking warnings impact applicant reactions.

**H_4_**: The Immediate Authentication Warning, Future Authentication Warning, and Moral Suasion Appeal will lead to different applicant reactions than a Traditional Faking Warning or No Faking Warning control group.

## Method

### Participants

We invited 535 military trainees from the Canadian Armed Forces to take part in our study. A total of 466 (87%) military trainees agreed to participate. Consenting military trainees provided their service numbers so that we could obtain demographic information and earlier test scores (e.g., cognitive ability) from their service files (*M*_age_ = 24.50, *SD* = 5.59, 88 females, 94.9% Regular Force, and 5.1% Reserve Force).

### Measures

#### Personality

We measured personality using two measures. The first measure, used during initial recruitment, was the Trait Self-Descriptive Personality Inventory (TSD-PI; Darr, 2011). The TSD-PI is the Canadian Armed Forces’ proprietary measure of the Big Five personality dimensions and has 75-items that examine Agreeableness, Conscientiousness, Emotional Stability, Extraversion, and Openness. Each item is measured using a 7-point scale (1 = “*Extremely Uncharacteristic”* to 7 = “*Extremely Characteristic”*). The TSD-PI is reliable *(αs* = .88 to .93) and each of the five dimensions correlate with their respective dimension on the NEO. Given that prior research that suggests military applicants are most likely to fake on Conscientiousness, Emotional Stability, and Extraversion, these are the three dimensions we analyzed in our study (Boss et al., 2015; Holden & Book, 2009).

We also measured the Big 5 personality dimensions using the 120-item version of the International Personality Item Pool (Goldberg et al., 2006; Maples et al., 2014) that uses a 5-point response scale (1 = *Strongly Disagree*, 5 = *Strongly Agree*). The 120-item scale has strong internal consistencies between .87 and .90 for the five dimensions. Additionally, all five dimensions on the 120-item scale were found to parallel their respective NEO Personality Inventory dimension with correlations between .87 and .90 (Maples et al., 2014).

#### Blatant extreme responding

We assessed faking using Blatant Extreme Responding. We calculated Blatant Extreme Responding by summing the frequency that trainees endorsed the most favorable response (i.e., *strongly agree* or *strongly disagree*) while engaging in our simulation. Thus, for each item, an trainee received a score of 1 (*extreme*) or 0 *(not extreme*), and we calculated the sum of those extreme responses. This approach differs from the Blatant Extreme Responding used by Landers et al. (2011), who assigned scores (0, .25, .5, .75, 1) for each level of the Likert scale (1 to 5, respectively) and then calculated the sum. In our opinion, one limitation of the Landers approach is that it is a transformation of the mean, and in turn, is logically conflated with legitimate personality scores. The main utility of measuring faking is to differentiate between those who are genuinely high on a trait and those who are not, so companies know who to hire and who to screen out. Our approach measures the tendency to answer with the most extreme answer, rather than just achieving a high overall score. This should lessen – but not eliminate – the chief limitation of the Landers approach to calculating Blatant Extreme Responding, which may flag as fakers the very people a firm wants to hire. Thus, we counted the frequency of extremely favorable responses across all five personality dimensions, where higher scores should reflect more faking. Blatant Extreme Responding has been used in multiple investigations and has been demonstrated as an effective measure of applicant faking (Landers et al., 2011; Levashina et al., 2014). We derived our Blatant Extreme Responding measure using the 120-item IPIP scale cited above.

#### Procedural justice perceptions

We assessed Procedural Justice Perceptions using three dimensions from the Selection Procedural Justice Scale (Bauer et al., 2001), which included Job-Relatedness, Information Known, and Chances to Perform. The composite scale had nine items that used a 5-point response scale (1 = “*Strongly Disagree*” to 5 = “*Strongly Agree”*). Example items for Job-Relatedness, Information Known, and Chances to Perform were “Doing well on the test means a person can serve well for the Canadian Armed Forces,” “I knew what to expect on the test,” and “I could really show my skills and abilities through the test,” respectively. The Selection Procedural Justice Scale has strong evidence of internal consistency (*α* = .88) and validity (Bauer et al., 2001). The scale also demonstrated construct and criterion-related validity (Bauer et al., 2001; McCarthy et al., 2013).

#### Test-taking anxiety

We measured Test-Taking Anxiety using the Comparative Anxiety subscale from the Test Attitude Survey (Arvey et al., 1990). Test-taking anxiety was measured using ten items on a five 5-point response scale (1 = “*Strongly Disagree,”* 5 = “*Strongly Agree”*). The Test Attitude Survey has evidence of internal consistency (*α *= .80) and criterion-related validity. The scale was previously validated using reactions to the Armed Services Vocational Aptitude Battery (Arvey et al., 1990).

### Procedure

We ran the experiment with 13 different groups in a classroom setting, with a range of 29 to 52 military trainees per class. We informed trainees about the study, stressed that participation was voluntary, and explained that their responses would not influence their military careers. Military trainees within each classroom were assigned to the same condition, where they received either the Traditional Faking Warning, Immediate Authentication Warning, Future Authentication Warning, Moral Suasion Appeal, or No Faking Warning personality test instructions (see Table 1). We used a random number generator to determine the order of the condition assignment and assigned our first ten groups to these condition in order. We then assigned the last two groups to conditions with smaller samples. We used this approach to minimize differences in group size. Still, we had an unequal distribution of military trainees across faking warning conditions (*ns*: Traditional Faking Warning = 83, Immediate Authentication Warning = 84, Future Authentication Warning = 54, Moral Suasion Appeal = 107, No Faking Warning = 114). In a couple of instances, military trainees joined the classroom too late to take part or were withdrawn during the simulation for military operations, creating unequal group sizes.

After military trainees were seated, we provided a general overview of the experiment, including why personality measures are important for recruitment decisions into the Canadian Armed Forces. To comply with ethics requirements, we minimized deception and informed all military trainees that the Canadian Armed Forces was looking to improve the accuracy of their personality test by piloting different sets of test instructions. However, we did not inform them of our hypotheses and the same script was used for all groups to ensure trainees were blind to the study’s manipulation.

**Figure 1.**
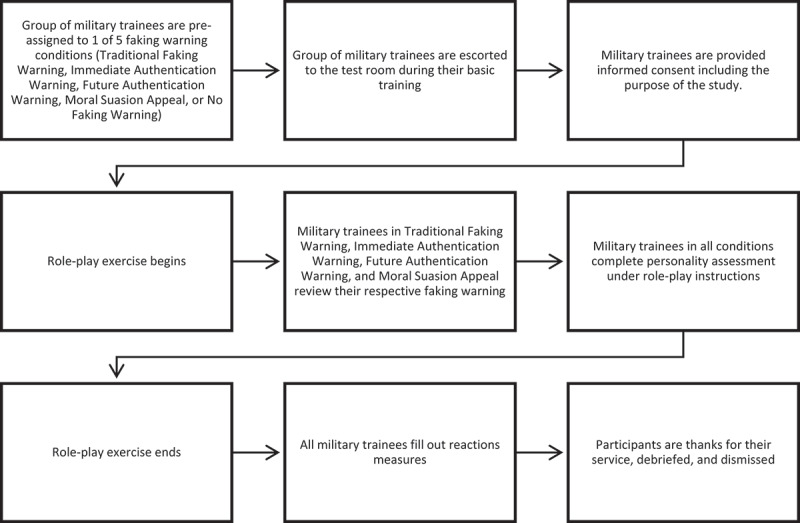
Procedure flow chart.

We instructed military trainees in the four separate warning conditions to engage in a selection role-play exercise, where they would fill out the surveys as if they were initially trying to secure employment with the Canadian Armed Forces. For each faking warning condition (Traditional Faking Warning, Immediate Authentication Warning, Future Authentication Warning, Moral Suasion Appeal, and No Faking Warning), the experimenter guided trainees through the study by displaying the role-play instructions and warnings on PowerPoint slides, which were displayed on multiple monitors. We used this approach to ensure that trainees were aware of the instructions before filling out the surveys and to keep them at a similar pace, so that we could better control the group. Next, these military trainees were asked to fill out our personality measures as if they were trying to be recruited by the Canadian Armed Forces. After completion, they were directed that the role-play was over and to fill out the remaining surveys honestly. Military trainees in the no-warning group did not engage in the role-play, were encouraged to respond as honestly as possible, and completed the personality test without the simulation. To encourage trainees to follow our instructions, we reminded them that their responses would be unassociated with their personnel file and would not influence their military service careers in any way. Trainees then completed a measure of procedural justice perceptions and test-taking anxiety. As before, the experimenter guided trainees through each measure – one at a time – using PowerPoint slides and reading instructions aloud. The experiment took 30 to 35 minutes per group. A procedural flow chart is provided in Figure 1.

The data from this study are property of Defense Research and Development Canada and are not allowed to be shared publicly.

## Results

### Preliminary analyses: Pre-experiment personality by condition

We did not have true random assignment, and as a result, we tested to see if mean personality scores varied between our faking warning conditions prior to our study. Group differences could provide alternative explanations for any differences between our faking warning conditions. To test for this, we conducted a MANOVA with faking warning condition as the independent variable and the three “Big Five” dimension scores (Conscientiousness, Emotional Stability, and Extraversion) on the TSD-PI during initial recruitment (prior to the experimental manipulation) as the dependent variables. There was no significant multivariate effect for faking warning condition, *F*(12, 1198.82) = 1.56, *p* = .10, Wilk’s *λ* = .96. This suggested that there were no differences in baseline personality scores across our faking warning conditions.

### Faking warning efficacy on IPIP

We examined the efficacy of our faking warnings by conducting a MANOVA with faking warning condition as the independent variable and Big Five personality scores from the IPIP as the dependent variables. We found a significant multivariate effect of faking warning condition, *F*(12, 1161.78) = 2.05, *p* = .02, Wilk’s *λ* = .95. The univariate results suggest that only the univariate test assessing the effects of faking warning condition on Conscientiousness was significant, *F*(4, 441) = 3.31, *p* = .01, η_p_^2^ = .03. Tukey’s B post-hoc tests revealed that trainees in the Immediate Authentication Warning condition reported significantly lower scores on Conscientiousness (*M* = 3.58) than did the trainees in the Traditional Faking Warning (*M* = 3.80), Future Authentication Warning (*M* = 3.76), Moral Suasion Appeal (*M* = 3.74), Traditional Faking Warning, and No Faking Warning (*M* = 3.77) conditions. No other differences were significant. Taken together, these results support **H_1_**, that the Immediate Authentication Warning would reduce applicant faking. These results provide no support for **H_2_** or **H_3_** that the Future Authentication Warning or Moral Suasion Appeal would reduce applicant faking. All means, standard errors, and 95% confidence intervals of the IPIP scores are presented in Table 2.

**Table 2.** Participant scores on the IPIP personality inventory during the application simulation.

Warning Condition	*n*	IPIP Personality Inventory									Blatant Extreme Responding		
		Conscientiousness			Extraversion			Emotional Stability					
		*M*	*SE*	95% CI	*M*	*SE*	95% CI	*M*	*SE*	95% CI	*M*	*SE*	95% CI
Traditional Faking Warning	87	3.80	0.05	[3.71, 3.89]	3.55	0.05	[3.45, 3.64]	3.59	0.05	[3.50, 3.68]	11.25	1.03	[9.22, 13.28]
Moral Suasion Appeal	113	3.74	0.04	[3.66, 3.83]	3.46	0.04	[3.38, 3.55]	3.54	0.04	[3.46, 3.62]	11.68	0.91	[9.90, 13.47]
Immediate Authentication Warning	84	3.58	0.05	[3.49, 3.68]	3.36	0.05	[3.26, 3.45]	3.47	0.05	[3.38, 3.57]	7.91	1.04	[5.86, 9.96]
Future Authentication Warning	57	3.76	0.06	[3.64, 3.87]	3.55	0.06	[3.42, 3.67]	3.69	0.06	[3.57, 3.80]	12.44	1.28	[9.92, 14.96]
No Faking Warning	118	3.77	0.04	[3.69, 3.85]	3.47	0.04	[3.39, 3.56]	3.53	0.04	[3.46, 3.61]	14.20	0.89	[12.45, 15.95]

This table displays the estimated marginal mean personality scores on the IPIP Personality Inventory and Blatant Extreme Responding during our experiment. Personality items were measured using a 5-point scale (1 = *Strongly Disagree*, 5 = *Strongly Agree*).

### Faking warning efficacy on blatant extreme responding

We also examined the extent that the faking warnings reduced scores on Blatant Extreme Responding. To do this, we ran an ANOVA with faking warning condition as the independent variable and Blatant Extreme Responding as the dependent variable. The results showed a significant main effect of faking warning condition, *F*(4, 458) = 5.39, *p* < .001, η_p_^2^ = .05. Tukey’s B post-hoc tests revealed that trainees in the Immediate Authentication Warning condition had significantly lower scores on Blatant Extreme Responding (*M* = 7.92) derived from IPIP scores on three of our Big Five dimensions than trainees in the Future Authentication Warning (*M* = 12.44), Moral Suasion Appeal (*M* = 11.68), and No Faking Warning (*M* = 14.20) conditions; but not the Traditional Faking Warning condition (*M* = 11.25). These results provide partial support for **H_1_**, as the Immediate Authentication Warning reduced faking compared to the no faking warning condition, but not the traditional faking warning condition. None of the analyses support that the Future Authentication Warning or the Moral Suasion Appeal reduced faking compared to the No Faking Warning or Traditional Faking Warning, suggesting that **H_2_** and **H_3_** were unsupported. Means, standard errors, and confidence intervals are reported in Table 2.

### Faking warning efficacy on trainee reactions

To test our final hypothesis, we examined the extent that the faking warnings influenced trainee reactions. To do this, we ran a MANOVA with faking warning condition as the independent variable, and Test Anxiety and three components of Procedural Justice Perceptions (Job Relatedness, Information Known, and Chances to Perform), as the dependent variables. The multivariate analysis did not approach significance, *F*(16, 1354.03) = 1.27, *p* = .21, Wilk’s Λ = .96. The results did not confirm **H**_4_ – faking warnings had no effect on trainee reactions.

## Discussion

We examined if faking warnings could thwart applicant faking among military trainees and if these messages engendered negative reactions. Our results provided support for our first hypothesis that the Immediate Authentication Warning would reduce faking relative to the Traditional Faking Warning and No Faking Warning. The Immediate Authentication Warning reduced personality scores on Conscientiousness relative to all other conditions and also reduced Blatant Extreme Responding when compared to all other conditions other than the Traditional Faking Warning. This suggests that the Immediate Authentication Warning reduces the likelihood that military trainees will respond with extreme responses (such as “7 = *extremely agree*”). These findings extend those of Landers et al. (2011) who found that faking warnings could reduce Blatant Extreme Responding in applied contexts. Moreover, the Immediate Authentication Warning was able to affect this reduction without accusing trainees of faking mid-test (as in Fan et al., 2012) and in turn, providing different test instructions to different participants. Thus, the Immediate Authentication Warning may facilitate some of the same gains can be had without the potential legal liability of providing different test instructions to test-takers, and in turn, disadvantaging one group relative to another.

It is important to acknowledge that these findings only provide partial support for the use of the Immediate Authentication Warning to reduce faking among future military applicants. In our study, the Immediate Authentication Warning provided a modest reduction in Conscientiousness scores relative to the no warning group (*Ms* = 3.58 and 3.77), and did not reduce scores on other personality measures. This suggests that the Immediate Authentication Warning, while producing a statistically significant reduction in faking, does not eliminate faking. The results showed larger reductions in Blatant Extreme Responding, where extreme responding was reduced by nearly half compared to the No Faking Warning condition (*Ms *= 7.90 and 14.20 respectively). However, the Immediate Authentication Warning did not perform significantly better than the Traditional Faking Warning in reducing Blatant Extreme Responding (*M* = 11.25), even though scores were in the intended direction. This suggests that the traditional faking warning may yield many of the benefits of newer and more elaborate messages. It also suggests that faking warnings are only so effective, and that practitioners and scholars alike should temper their expectations when using faking warnings to combat faking in applied contexts. We expected the extra threat of verification in the Immediate Authentication Warning to influence test reactions among the trainees but found little evidence of increased negative reactions or lower ratings of procedural justice when compared to the other warning conditions.

There are two potential explanations for why the Immediate Authentication Warning did not produce more adverse reactions. First, and in accordance with Goffin and Boyd’s (2009) faking decision tree model, applicants go through a series of decisions when choosing to fake on each test item, such as considering the morality of faking, the chances of detection, or whether faking will lead to disqualification. The Canadian Armed Forces military trainees may have experienced the same decision-making process during the application simulation, regardless of their condition, and as a result, the same cognitive burden or anxiety across faking warnings. Second – and more concerning – is that the trainees may not have experienced negative reactions because we had them engage in a simulation without real consequences for their career. However, their responses are more in-line with what we expect from organizational faking – faking on the most important personality dimension and reductions in the most obvious form of faking. One might expect a simulation and demand effects to cause more extreme and less discriminant faking.

While the Moral Suasion Appeal and Future Authentication Warning failed to thwart applicant faking, the null effects still meaningfully contribute to the field. Several scholars have postulated that “softer” faking warnings that appeal to morality, educate, and have applicants consider long-term consequences will reduce applicant faking – rather than threaten applicants (Goffin & Boyd, 2009; Uruena & Robie, 2011). The results of our studies do not support this speculation. In addition, we found that a faking warning, which capitalizes on short-term accountability and immediate consequences, can be more effective than traditional warnings, yet do not engender negative reactions in simulated application scenarios. Together, these findings provide no incentive to further investigate these “softer” messages. Instead, future research on faking warnings should try to maximize short-term accountability by implementing more compelling descriptions of how faking can be detected. Our findings suggest that faking warnings may be unable to convince applicants to consider the morality of faking, or to be more concerned about the long-term consequences of faking beyond their dispositional inclinations.

Our findings suggest the Moral Suasion Appeal and Future Authentication Warning were ineffective in preventing faking, as other factors are more important to the decision to fake. Based on accountability theory, we expected that trainees in the Future Authentication Warning condition would worry about the long-term potential of verifiability and the need to defend their answers during a one-on-one observation; clearly, our findings do not support these ideas. We entertain two potential explanations for this finding. First, that trainees believed that the short-term reward of securing employment in the Canadian Armed Forces simply outweighed the risks of being detected at a future time. The trainees may also have believed that they could emulate the personality they portrayed, and in turn, genuinely viewed it as low-risk to fake (Goffin & Boyd, 2009). Second, as discussed earlier, military trainees participated in a simulation and may not have been able to imagine the scenario as they would experience in a real job application. However, this is unlikely, as most trainees had applied for a position within the past year at the Canadian Armed Forces, and therefore, should have been able to imagine the application scenario. Additionally, job applicant simulations with real employees tend to provide realistic estimates of faking when compared to student samples (Goffin et al., 2011).

We also found that moral prompts about the negative outcomes of faking did not reduce faking by trainees. Goffin and Boyd (2009) suggest that the morality of faking is the first decision that applicants consider when answering personality items during the hiring process. It is possible that applicants may not consider morality first in the decision-making process; instead, applicants may believe that faking is normal. Indeed, the majority of applicants do fake (Donovan et al., 2003; Feeney & Goffin, 2015; Holden et al., 2017).

Another possibility is that there may be external factors that are too powerful for test instructions to override. For example, if an applicant is applying for a position when they do not have enough money to support their family, they may view faking as a lesser evil than not providing for their family. Thus, morality may be relative to the applicant’s need for employment and external considerations. If this is the case, then test instructions appealing to morality are unlikely to be effective. Similarly, the moral consideration of applicant faking is dispositional rather than situational. For example, we know some people are higher in trait integrity than others, and that these traits predict workplace delinquency (Lee et al., 2005). Therefore, virtuous applicants may be unlikely to fake, regardless of the test instructions, whereas others may not care about the morality of faking. Unfortunately, due to time constraints, we were unable to ask the military trainees about their reactions after the simulation.

## Limitations

The main limitation of our study is that we used a recruitment simulation with the military trainees, and in turn, their results may not generalize to actual applicants experiencing a high incentive to perform in a real scenario. We used a simulation because both the Canadian Armed Forces and our university comply with national ethics policies from the Canadian Social Sciences and Humanities Research Council, which would forbid administering different test instructions to real job applicants, as some applicants may be disadvantaged relative to others as a function of our research. It was paramount that participants’ futures, or possible careers, not be affected by the experimental outcomes that might reward applicants in one condition, while punishing applicants in another condition. Therefore, every effort was made to re-create the initial application process (Aguinis & Bradley, 2014). Future research may be able to balance ethical concerns and enhanced experimental design by incorporating the faking warning study during the application process after applicants complete the standard assessments used for hiring, thus allowing an employer to make their hiring decision based on non-experimental data.

The second limitation is that the military trainees were assigned to us in large groups (29 to 52) and we assigned groups (rather than individual trainees) randomly to our different experimental conditions. We also had to defer to military operations in some instances, which may have confounded our assignment. For example, our smallest group had several military trainees removed in the early stage of our study for administrative purposes. The groups also had substantial variations in reading speed, and for logistical reasons, we moved at the average pace of trainees. This led some trainees who were behind to skip sections or submit their package prematurely – which may have produced some systematic bias, especially for survey questions at the end of our study. Finally, we assumed that all trainees paid sufficient attention to and internalized instructions for each of the manipulations; however, findings suggesting the relative effectiveness of Immediate Authentication Warning condition in preventing faking provide partial evidence of the manipulation’s success.

## Implications and conclusions

Our findings have two primary implications for researchers and human resource practitioners. The first implication is that faking warnings that emphasize short-term accountability are the most effective at combatting applicant faking. This effect is best utilized by making applicants believe that there is a credible process to verify their answers, such as threatening to check applicant responses with personal references they provided prior to the personality test. Future research will need to explore new avenues to improve the efficacy of faking warnings that result in immediate removal from the selection process. Future studies should also examine whether new warnings help guide applicants to engage in more sophisticated faking behavior. This behavior can be directed toward avoiding detection. Further, researchers should consider the psychometric consequences of the repeated use of these faking warnings. For example, faking warnings lose their efficacy with repeated administrations to the same applicants (Landers et al., 2011). The second implication is that there is little incentive to continue investigating “softer” faking warnings, which emphasize educational or moral suasion. These warnings may not appreciably reduce faking and may lead to more positive applicant reactions compared to more threatening messages.

## Data Availability

The data from this study are property of Defense Research and Development Canada and are not allowed to be shared publicly.
